# 

**Published:** 1955-11

**Authors:** 


### THE MEDICAL JOURNAL OF THE SOUTH-WEST

*Incorporating*
*The Bristol Medico-Chirurgical Journal*

ARMED FORCES
DEC 28 1955
MEDICAL LIBRARY

*November 1955*

